# 601 Potential Clinical Signatures for Early Inhalation Injury Prognosis in Burns Patients

**DOI:** 10.1093/jbcr/irae036.235

**Published:** 2024-04-17

**Authors:** Tarryn K Prinsloo, Wayne G Kleintjes, Kareemah Najaar

**Affiliations:** Cape Peninsula University of Technology/Biosigns Therapeutics, Cape Town, Western Cape; Stellenbosch University, Cape Town, Western Cape; Cape Peninsula University of Technology, Cape Town, Western Cape; Cape Peninsula University of Technology/Biosigns Therapeutics, Cape Town, Western Cape; Stellenbosch University, Cape Town, Western Cape; Cape Peninsula University of Technology, Cape Town, Western Cape; Cape Peninsula University of Technology/Biosigns Therapeutics, Cape Town, Western Cape; Stellenbosch University, Cape Town, Western Cape; Cape Peninsula University of Technology, Cape Town, Western Cape

## Abstract

**Introduction:**

Despite being one of the most important mortality co-factors; inhalation injury is complex and diagnosis remains variable with limitations, especially in a resource-constrained clinical setting. Potential relationships between inhalation injury and injury-related factors were investigated that could provide inferences suggestive of improving early prognosis in burns patients in the absence of diagnostic tools.

**Methods:**

This cross-sectional, cohort study commenced following ethical approval and patient consent. Clinical information was collected from burns patients (n=59) admitted to our Burns Centre between 23 April 2016 and 15 August 2017 irrespective of etiology. The primary outcome was inhalation injury based on the patients’ total ventilation days and stratified into mild and severe (≥5 days). Parameters included sociodemographic (gender, age, etiology), burn severity (%TBSA and complications), and clinical variables (burns intensive care unit length of stay-BICU LOS, partial gas pressures, and lactate). Descriptive statistics (mean (95% CI) and/or frequency (%)), the Spearman Rank coefficient (rho) for correlation strength/direction, and partial least squares regression for variable of importance in prediction (VIP) values, were performed. Statistical significance was denoted by p< 0.05 (two-tailed) and analyses were performed using IBM SPSS Statistics version 28.

**Results:**

An average of 2.7 patients (95% CI 2.3-3.4) were diagnosed with mild inhalation injury and 11.2 patients (95% CI 9.5-12.9) with severe inhalation injury. The latter was associated with a 93.3% mortality rate. Positive values were observed with all significantly correlated factors which ranged in strength from moderate (%TBSA rho=0.357 and lactate rho=0.331) to strong (complications rho=0.690) and very strong (BICU LOS rho=0.908). Complications (VIP=1.229) and BICU LOS (VIP=1.372) had the highest VIP values in the regression model.

**Conclusions:**

Inhalation injury degree positively correlated to the presence or progression of the assessed parameters, with complications and BICU LOS observed as the best predictors in the current model. Initial in-hospital prognosis could benefit by considering the factors that would immediately present on admission or shortly thereafter. Complications may therefore be more suitable for the latter, whereas, increased BICU LOS could rather provide insight into degree progression.

**Applicability of Research to Practice:**

Identifying the clinical parameters that would improve timeous treatment of inhalation injury could in turn reduce the exacerbated inflammatory state of burns patients and subsequent mortality likelihood. These findings suggest that increased inhalation injury presence and/or degree should at least be considered when these factors are present/increased.

